# Social workers’ motivation to provide effective service in a challenging environment of illegal substance abuse

**DOI:** 10.4102/hsag.v31i0.3231

**Published:** 2026-04-22

**Authors:** Oratile Khampepe, Nathaniel P. Kgadima

**Affiliations:** 1 Department of Social Work, College of Human Sciences, University of South Africa, Pretoria, South Africa; 2 Department of Social Work, College of Human Science, University of Pretoria, Pretoria, South Africa

**Keywords:** care, compassion, effectiveness, gratification, motivation, self-fulfilment, support

## Abstract

**Background:**

The social work profession is portrayed as a stressful occupation that brings about depression and demoralisation upon social workers. It is believed that working towards changing an individual, group or community at large is difficult.

**Aim:**

To explore the gratification that social workers receive from working with youth abusing substances.

**Setting:**

Data were collected from social workers employed by the Department of Social Development, from both the Sebokeng and Vereeniging offices, as well as the South African National Council on Alcoholism and Drug Dependence in the Vaal Triangle area.

**Methods:**

This qualitative and phenomenological study was undertaken with a sample of 15 participants who were selected through purposive sampling. Data were gathered through semi-structured interviews and analysed thematically.

**Results:**

The study highlights the significance of creating a supportive work environment to maintain good performance and well-being amongst social workers in the substance abuse or misuse section.

**Conclusion:**

Many studies record the struggles that social workers endure when rendering services to the youth, adolescents and other groups abusing illegal substances; yet some find gratification when rendering services, which is not recorded.

**Contribution:**

This study will be of use to students at universities in knowing that there is fulfilment, as well as professional and personal development, in working as a substance abuse social worker. A great contribution will be made to the employer in knowing that providing tools of trade empower social workers professionally and personally, and enable them to render services smoothly, finding gratification whilst doing it.

## Introduction

### Background

Social workers play a crucial role in supporting individuals struggling with substance abuse. According to Sapiro and Ward (2020), social workers are an important resource in helping at-risk youth to get well, providing them with emotional support, and putting them in touch with the necessary services. However, social work has become a stressful and difficult profession where social workers increasingly experience burnout and trauma, together with other work-related stress illnesses that affect their well-being and ability to assist clients (Senreich, Straussner & Steen 2020). Although treating addiction can be a difficult activity, seeing progress – whether it be in the form of minor behavioural adjustments, stronger family ties or effective rehabilitation – brings a profound sense of fulfilment and purpose for many social workers (DiClemente 2018). Positive emotional and cognitive outcomes brought on by caring for others are referred to as compassion satisfaction (Hansen et al. 2018). These outcomes include feeling better about oneself, content with one’s circumstances, growing personally and feeling reinforced by having been able to assist others. Other authors (Bae et al. 2020) found that when social workers experience compassion satisfaction, they frequently report higher levels of satisfaction with their employment, which can lead to improved professional practice.

This study highlights that the self-fulfilment of social workers adds to the little, or non-existent, positive literature on substance abuse social workers rendering services to the youth. Many studies have recorded the struggles that social workers face when rendering services to the youth, adolescents and other groups abusing illegal substances. However, some find gratification when rendering these services, which is not recorded. The literature will be of use to students at universities in knowing that there is fulfilment, as well as professional and personal development in working as a substance abuse social worker. Furthermore, a great contribution will be made to the employer in knowing that playing their part and providing tools of trade to employees, managers and supervisors, providing support to their subordinates in return, empowering social workers professionally and personally, and enabling them to render services smoothly and find gratification whilst doing it.

### Research question and problem statement

The primary, overarching research question of the study was: *What motivates social workers to provide effective service in a challenging environment of illegal substance abuse?*

Many studies explore the challenges faced by social workers in every field of the social work profession and how they struggle to render services, accompanied by health issues related to the work environment. Treating substance use disorders in communities and clients can be extremely difficult. The engagement process is the first obstacle. However, regardless of the challenges this job presents, there is also evidence that social workers may find their function fulfilling and feel satisfied with their work, believing that they are making a significant difference in people’s lives (McFadden, Campbell & Taylor 2015). Despite its fundamental importance in the profession, limited studies seem to have been conducted addressing compassion, specifically within the context of social work practice (Clark & Jen 2023). As social workers deal with stressful situations at work, this feel-good article will hopefully rejuvenate their morale when facing challenges. When social workers are compassionate, they are likely to be more productive.

### Theoretical framework

The study employed the Human Caring Theory by Jean Watson (1979, revised in 1985 and 1988). Initially developed in nursing scholarship, the key foundation of this theory was caring for the self and others (Montana-Rhodes, Kennedy Oehlert & Hill 2024; Murali 2020). Watson (1979, revised in 1985 and 1988) purports that the purpose of caring is to assist people in achieving a greater degree of harmony between their mind, body and spirit, which is a sign of health, and that caring is a desired ethic that eventually leads to the maintenance and advancement of human dignity (Gunawan et al. 2022). Furthermore, Watson (1979, revised in 1985 and 1988) declares that the fundamental idea of Human Caring Theory is that people cannot be viewed as objects and that they are inseparably linked to their selves, others, the natural world and the cosmos (Ravi 2018). The foundational assumptions of the research investigations argue that the principles of human caring are universal and prevalent across several professions (Wei & Watson 2019). Therefore, social workers in the substance abuse field are viewed as a source of support by patients with psychoactive substance addiction issues, assisting them in managing their pain and severe emotional experiences (Curcio et al. 2024).

## Research methods and design

### Research goal, approach and design

This qualitative and phenomenological study was deemed relevant to explore the gratification that social workers receive from working with youth who abuse substances. Phenomenological research studies attempt to discover and describe people’s lived experiences to have a deeper understanding (Cilesiz 2011:492).

### Population

Using the purposive sampling method, this study reached data saturation with 15 social workers employed by the Department of Social Development (DSD), from both the Sebokeng and Vereeniging offices, as well as the South African National Council on Alcoholism and Drug Dependence (SANCA) in the Vaal Triangle area.

The main criteria for inclusion were that the participants should be registered as social workers in terms of the relevant South African legislation and that they should have been practising in the field of substance abuse for a period of at least a year and more.

### Data verification

To ensure credibility, the researcher shared the recordings of the participants’ interviews, as well as the results of the study with the participants to assure them that the researcher had not altered their words. Regarding transferability, the researcher interviewed social workers from different offices, but those working in the substance abuse section. Dependability was ensured by the researcher and supervisor auditing the work to determine good practice, and the final work was made available to the auditors to determine the validity of the conclusion reached. Confirmability was achieved by the researcher by allowing the participants to express their views without any interference. The data were then transcribed, and the findings compared with the existing literature.

### Data collection and analysis

Data were collected through face-to-face semi-structured interviews. The first part of the interview schedule included the participants’ biographical profile, as indicated in Table 1. The topics covered in the second segment of the interview schedule translated into the four themes below, namely the participants’ ‘motivation for working with service users’; the participants’ ‘passion towards their works’; the participants’ ‘appreciation of support from the managers and/or supervisors’; as well as the participants’ ‘appreciation of available resources to fulfil their duties’. All the interviews were mainly conducted in English as all the participants were conversant with language. Only in some instances where some participants used indigenous languages to explain some concepts. The intention of translation process in this study was for meaning rather than literal equivalence. The authors also relied on their knowledge and understanding of the participants’ indigenous languages to provide a comparable translation and interpretation.

**TABLE 1:** Biographical information of the participants.

Participant’s code	Gender	Age (years)	Race	Home language	Years’ work experience as social worker
1	Female	37	African person	Sesotho	9
2	Female	29	African person	Sesotho	9
3	Female	47	African person	isiZulu	4
4	Female	31	African person	Sesotho	8
5	Female	48	African person	Sesotho	4
6	Male	35	African person	Sesotho	8
7	Female	46	African person	Sesotho	6
8	Female	45	White person	English	3
9	Male	28	African person	isiZulu	6
10	Female	34	African person	Sesotho	7
11	Female	46	African person	isiXhosa	8
12	Female	37	African person	isiZulu	1
13	Female	49	African person	Sesotho	4
14	Female	30	African person	isiZulu	6
15	Female	30	African person	Sepedi	7

The data were analysed thematically. The data analysis process included transcribing and reading the recorded data, followed by thematic analysis until themes were identified and categorised.

### Ethical considerations

Ethical clearance to conduct this study was obtained from the University of South Africa College of Human Sciences Research Ethics Review Committee on 31 January 2022. The ethical clearance number is 69290628_CREC_CHS_2022. The following ethical considerations were observed, namely obtaining written informed consent, assuring confidentiality, protecting participants from harm and managing the research data. With regard to the ethical principle of informed consent, the participants were furnished with information regarding the purpose of the study process, the risks and benefits associated with participating in the study. The ethical principle of confidentiality was adhered to by not revealing the participants’ identities, but instead reporting the data anonymously by using pseudonyms. Upon completing each of the interviews in the study, the participants were informed that a debriefing service was available if the interview triggered any negative emotions. However, all the participants expressed no need for the debriefing service.

## Results

The findings from the interviews conducted with the 15 participants are presented below. The data received were transcribed, coded by an independent coder and analysed thematically using existing literature.

### Biographical information of the participants

Table 1 presents the biographical information of the participants in this study. Indicated in this table are the participant’s code, gender, age, race, years’ experience as a social worker and the year in which their degree was obtained. The home language and office of employment of the participants are also included.

Considering that social work has long been regarded as a non-traditional field for men, it was expected that the study would include more females than males. As this study was conducted in a predominantly black community around the Vaal area, only one white participant was interviewed, with the rest being black African people across different ethnic groups. Most of the participants far exceeded the inclusion criteria of minimum years’ social work experience.

### Presentation of themes

#### Theme 1: Participants’ motivation for working with service users

Many social workers are drawn to the field by their altruistic desire to improve people’s lives and society, as well as their compassion for others (Decker et al. 2015:28). Several factors influenced their motivation, such as the passion they have for working with people, changing people’s lives for the better and receiving positive feedback from the family of the addicted. Some are also willing to change to other sections for the sake of growth within the department.

Men seem to use drugs more frequently than women. A study conducted by Peltzer and Phaswana-Mafuya (2018:5) about drug use amongst youth and adults in a population-based survey in South Africa found that men were substantially more likely to use drugs than women. Hence, one participant raised the desire to save the next generation of men from substance use and create a better future for them. The following extract attests to this:

‘I would say first, the youth are the future of our country and seeing so many guys, especially because that there are more males than females using substances. It is a shame to see so many men drowning in substance use, because it kills the future generation of our country. The passion is there because once you save one person from illegal substance use, it gives us hope that at least there is one less guy abusing drugs, meaning that there is one father for their family and their future.’ (P10, female, 34 years)

Authors argue that a person who has a passion for something has a strong predisposition towards it and invests time and effort into it (Yudaruddin et al. 2022:17). One of the participants considered becoming a social worker being a dream come true, which is indicated in the following response:

‘As a young girl, it was my dream to become a social worker. So, I had the privilege of staying at home with my kids doing everything with them, and now they are grown, and I decided to follow my passion and registered for social work. So, I am so passionate, it’s my dream, it’s a calling, let me put it that way. Helping people makes me feel great about doing my job.’ (P13, female, 49 years)

Another participant appreciated the importance of raising awareness amongst people about substance use. The participant’s appreciation is captured in the vignette below:

‘You know when you get to make people realise things that they did not clearly identify as a problem … like I am saying now, lots of parents would accept and express that “you know what, I never saw it that way, I never viewed it in that manner.” Most of them will tell you “yes” when you ask them “have you ever thrown a party for a one-year-old?” or “have you been invited to one where the party concentrated only on kids having fun, or later, after the hip-hip hooray you are now having adults’ party?” So, that gives me the passion to want to meet more people and speak about these things. And to tell them that it is a mistake, you did it because you did not know, you know.’ (P1, female, 37 years)

Social work is known as a profession changing the lives of people. Underpinned by theories of social work, social sciences, humanities and indigenous knowledge, social work engages people and structures to address life challenges and enhance well-being. Similarly, some participants shared their motivation and passion for working with the youth and changing their lives. They further shared about the gratification that emanates from achieving their goal. The extract below elaborates further:

‘My motivation is the transformation that we see in the people that we help, the new lives. And if I can just elaborate beyond that, when you come back from the rehabilitation centre to fetch your clients, I always prefer to, but some social workers don’t like to fetch clients who have graduated and then completed their programmes. But I prefer to do that because I like to see the reaction on their family’s faces, it is very emotional, but now the emotions are different; it is not the sad emotions. It is the tears of joy, you know, and of being very thankful, and they are shocked, surprised that they could never believe that they could see this person in the state that he or she is in you know.’ (P6, male, 35 years)

A small number of service users put in the effort to change their behaviour of substance use. Despite that small number, change has been seen, which motivates the participants. This brings about pleasure to the participants. The extracts below attest to this:

‘There are people who do not quit, then you get motivated to say, “at least there are positive stories and there are also not so positive stories that we are facing.” But seeing your client beautiful, clean and working, that is motivating, [*expressing a happy facial gesture*] yho! [*exclaims*], it is motivating.’ (P8, female, 45 years)

‘So, even if they are relapsing, nothing motivates me like seeing one person being sober. I think that motivates me because I think I may, ahh, I think am always saying at least there is someone who sees like my assistance out there … I think I always tell myself that you know what, I made a difference in that family or in that person’s life. One will be able to wake up and go to aftercare services and you know one of my clients is working with … he is at one of the rehabilitation centres as a security guard. Nothing motivates me more than that.’ (P11, female, 46 years)

One of the participants shared that they are motivated by the feedback they receive from parents and their clients to continue working in the substance use section. The participants’ appreciation is captured in the following extracts:

‘I am telling you … I am more passionate, regardless of the problems, the challenges that we are facing when working with substance use. But the feedback from the parents, the feedback from the clients, they are the ones motivating me to be more passionate.’ (P3, female, 47 years)

‘I don’t know, but I think I’m passionate because, for example, if the parent comes to the office with a child, obviously who is using drugs, they will just think “no, this child is silly or whatever.”’ (P2, male, 29 years)

The storylines revealed that positive feedback from parents creates motivation for participants to continue assisting clients. Devaney et al. (2023:487) note that it has been discovered that parents who receive services are aware and appreciative of practitioners who ‘go the extra mile’ for them.

Some participants tried their best to deliver services to their clients, irrespective of circumstances. This is done, regardless of whether clients play their part. The following extract attests to this:

‘The motivation is trying, at the end of the day, that is all that we can do. I cannot help the client fully, especially if they don’t participate, so the fact that I know that I have done my part; that is the motivation. This is what I must do to support the clients, but the clients also must play their part. The family also must play their part. So, whether the client is surviving or not, it is not up to me. At the end of the day, I played my part and luckily, I have only two, but it is a work in progress.’ (P4, female, 31 years)

The extract seems to suggest that participants come across different challenges when assisting clients, but nonetheless, they proceed with rendering services. Similarly, no matter where social workers carry out their tasks, they encounter some universal difficulties (Khanyi & Malesa 2022:31). However, it puts them at ease knowing that they have played their part.

#### Theme 2: Participants’ passion for their work

Despite the low salary received by social workers compared to the number of services they deliver to their clients, some participants are still passionate about their work, as these extracts attest:

‘I want to make a difference, that’s the only reason I think you would know that social workers are underpaid [*giggles*], but the passion needs to keep you going. There’s a friend of mine who always jokes ugithi thina si sebenzela ipassion [*we’re just working for passion here*] more than anything. So, being passionate, making a difference as well.’ (P12, female, 37 years)

‘The motivation, I think it’s the love and the passion. The not-good-pay part because we are not being paid like nicely, not getting good salary, but the passion is forcing me, it is what’s keeping me going every day because I like helping people and I love working with people. I am an extrovert, I love talking to people, I love working with people so … [*silent*]. It’s the working with people part that I love most.’ (P13, female, 49 years)

The findings reveal that social workers stay in their substance use programme only because of the passion they have towards saving the lives of their clients. Other social workers opt to move out of the country to seek better-paying social work jobs. A study about the experiences of South African social workers in the United Kingdom (UK) reported that they were unhappy with the service environment. They chose to work in the UK as they could make money in pounds, enjoy good working conditions and receive a higher salary (Naidoo & Kasiram 2006:120).

#### Theme 3: Participants’ appreciation of support from managers and/or supervisors

Having resources to deliver services makes it easy for social workers to appreciate doing their work and continue changing lives. The support of managers and supervisors plays an important role in the day-to-day service delivery of social workers. It is noted that their support through supervision ensures accountability and support with difficult cases, which are then dealt with more easily, and that there is professional development (Manthorpe et al. 2015).

Most participants shared positive experiences regarding the support they receive, with the extracts below attesting to this:

‘I would speak personally with the work I do [*pointing at herself*], my supervisor and manager. I would like to think that they offer me great support to achieve my daily work. You know they motivate us. Our supervisor and manager are always trying to help whenever we need them, but I do not know of other colleagues from other units.’ (P1, female, 37 years)

‘My manager and supervisor are on the roll. They do support us, hey! When you have a case, we get ministerial inquiries or there are cases from the province, our manager will be there, step by step with our supervisor until the case is solved, so they are always there; they are very supportive. I don’t want to lie. They guide you even when it’s a difficult case, they are there. The relationship is very good and professional, you can just feel free and know that you know what, I have a good support structure at work, so they are there all the time they are there.’ (P15, female, 30 years)

A healthy working environment is important to social workers owing to the type of services they deliver. Employees need a working environment that allows them to work freely and that is free from obstacles that can prevent them from achieving their full potential to satisfy the standards of the organisation (Raziq & Maulabakhsh 2015:718). The participant below expressed her healthy working environment further:

‘I think in as much as I have mentioned some of the flaws that the supervisor or manager may have, but it’s a very healthy working environment and I’m able to approach my supervisor when I have a pressing case and at times, yes, she is able to give me attention and guide me, and she’s someone who’s open as well. The same goes for the manager, so I’m not uncomfortable with the working relationship, they just need little improvement for example to say managers manage your staff better so I wouldn’t want to change any of them.’ (P12, female, 37 years)

A supervisor can assist subordinates by providing constructive performance reviews, career guidance, networking opportunities and job direction. Below, the participants stated the following regarding the support emanating from their supervisors:

‘Okay, once a month, we do have the supervision with our supervisor so that we can talk about our challenges. The main challenge that we have is people who have used substances and now are not well mentally. It is a challenge to us because the parents don’t understand, it is like, “No, I don’t want to take my child to the rehabilitation centre, you don’t want to help my child” … Yes, we do get support from the supervisor and sometimes you don’t know what to do as a social worker. Then we go to her, then she would advise us on what to do. In other cases, she would tell us what to do, like “this one is not for you, refer.”’ (P8, female, 45 years)

‘The supervisor refers us for trainings and renders supervision. I can say those are the support they provide for us to render our services. Yes, individual supervision with the supervisor and we used to have group supervisions where we would sit with colleagues and supervisor and share experiences of our clients. But, due to COVID-19 now … [*silent*] It had to stop. I think it is this COVID-19 pandemic that has caused this thing, because now there is also limited time because sometimes, we work remotely.’ (P3, female, 47 years)

Sound procedures, orientation and training have been found to yield positive results within a working environment. Training and orientation assist organisations in gaining and retaining top talent, increasing job satisfaction and morale, as well as improving productivity (Rabbani et al. 2017:3). The importance of training and orientation mostly revolves around programmes that enable employees to learn precise skills or knowledge to improve performance (Talukder, Vickers & Khan 2018:727). It is, therefore, critical that orientation and training on processes be given to employees to enhance their productivity. In supporting these views, one participant had this to say:

‘I think with the supervisor and manager, the relationships are good, because they are there for us and they understand that we are new in the profession, so we cannot master everything. So, they take us through each step of the way. We also sometimes have internal trainings whereby they call us together; we discuss our cases, and with the new people they come together and discuss … ok! This is what the process is in terms of substance use treatment, the procedures are this and this and this, and if you don’t understand, they assist you from there and the manager also, we do have debriefings if you feel that you have a serious challenge and the supervisor cannot assist, she can also refer you to the manager and then you discuss everything.’ (P14, female, 30 years)

It was established that social workers go as far as consulting the Chief Executive Officer (CEO) to solve their operational problems. One participant said:

‘We have an open relationship with our CEO. So, I know that I can just go to the CEO’s office, because there is an open policy. If I have a problem, I would speak to my supervisor first, most of the time before we go to the CEO to solve our problems. But most of the time, it does not go to the board members; it does not get that far. It will end up in the level of CEO.’ (P8, female, 45 years)

It has also been found that board members are providing support. One participant indicated the following:

‘The board members are supportive. We have nice support; I think the board members as well, because I am the staff representative. That is the only reason I attend the board meetings is because I am the staff representative of the board. Occasionally, I think the board that we have is exceptional[*ly*] supportive.’ (P7, female, 46 years)

It is evident from the storyline above that the participants from SANCA have the full support of their board members in the delivery of their services. They are responsible for providing strategic direction and oversight for the company, as well as ensuring that the organisation complies with all the relevant laws and regulations (Msuya & Kumar 2022:11).

#### Theme 4: Participants’ appreciation of available resources to fulfil their duties

Some participants acknowledged the availability of resources and commended their respective employers for their efforts in making resources available. Resources enable employees to complete their tasks and goals successfully and enhance their well-being and capacity to perform well. In the desire to drive social workers’ well-being, organisational growth and service delivery, there has been an increasing interest in resources at work.

**Availability of more social workers:** According to Madisha and Skhosana (2022:443), the existence of enough qualified substance use counsellors and social workers in South Africa with the prerequisite training, experience and skill has remained a primary benefit in substance use treatment. In validating this claim, the following extract was provided by a participant:

‘Okay, to start with, I remember when we started with this programme [*substance use section*], it was only four social workers. So now, there are almost 18 social workers. So, at least the number is growing, and we can manage our own caseloads.’ (P2, female, 29 years)

The participant above highlighted an increase in social workers in the substance use section. Such an increase has led to the proper management of caseloads, as well as more manpower to address the substance use crisis. Similarly, authors claim that social workers and addiction counsellors play a critical role in minimising the substance use epidemic through treatment and recovery (Filges & Jorgensen 2018:363).

**Availability of transport:** Transport remains a necessity in all public service departments for employees to reach their clients. The case is no different within social welfare organisations, because social workers have to move around in their areas of service to solve cases and to transport service users. Based on that, the participants stated the following:

‘The resources, I think government is doing their utmost best that we are able to use vehicles the whole day for whenever you want to go or transport your client for whatever reasons, as long as it is in your scope of practice. So, I think that makes our job to be easy as a social worker.’ (P11, female, 46 years)

‘So, I think government does quite alright, and we need to give them a hand there. They are providing resources; we do have vehicles to transport families who cannot afford to go to rehabilitation centre. We do family reunification services and now we do have telephones … cellphones from the employer and now when you are working, serving remote areas where there are no landlines, and we do have a lot of support. I think when it comes to resources, I cannot complain. And when it comes to development in terms of training, also there is support. I mean, I have attended a number of trainings. There is one I did not so long ago, I think it was 2020. Government is trying to get everybody on these streams, more especially on substance use disorder and it is a very updated training with modern research, you know.’ (P6, male, 35 years)

Some participants emphasised the importance of information sharing as many people still do not have enough information about drugs. Similarly, in a social welfare organisation, pamphlets and posters describing different illegal chemical substances and their resultant consequences should be available to ensure that consultation is effective, and that service users are well informed (Geyer, Le Roux & Hall 2015:322). The availability of printed material plays a big role in the service delivery of social workers. Information needs to be shared with clients and the community at large. One of the participants said the following in this regard:

‘So, there is a lot of consultation, you read our pamphlets, you see that government is really trying to be on top of their game, if you get what I am saying. Even our pamphlets they do describe what is nyaope, Nyaope is not something that was there many years ago. You know it popped up very recently so immediately we defined it. A lot of people still don’t know what nyaope is. They don’t even understand what the concept is, but I am happy with the trainings we are receiving. And in terms of debriefing, that is where I feel like I am getting a little bit of dissatisfaction with the employer. I also cannot really blame anyone, but I think it has to do more with time management from social workers and supervisors, we struggled to find time to sit and have case conferences and proper supervision that will help us when we are out there in the field and form part of debriefing. So that is an area that I feel it needs improvement.’ (P6, male, 35 years)

**Availability of rehabilitation centres:** The participants noted the availability of rehabilitation centres, which play a key role in the sobriety of their clients. A rehabilitation facility affords drug addicts an opportunity to learn from their mistakes and change their behaviour. Some of the extracts recorded are presented below:

‘Okay, I will start with what I am satisfied with, and it is resources. I think our department [*Department of Social Development*] is trying very hard, it is not easy. I think the treatment for substance use disorder is very expensive. But, since I am in the programme, I have seen more rehabilitation centres, I have seen more outpatient treatment centres popping up, I have seen more skills development centres open to support the whole process of treatment.’ (P6, male, 35 years)

‘With regards to rehabilitation services, yes, we do have them. We have, I think, uhmm, how many rehabilitation centres, let me see, the one in Jamela in Bophelong. Then we have Freedom Recovery Centre in Nigel, we have West View and Golden Highway in Randburg. We have Dr Fabian and Florence Ribeiro Treatment Centre Sick Bay and Admissions [*Dr F and F*], which is in Pretoria, and we have SAICA Horizon in Alberton [*using hands to count*]. We have the skills development centres that we work with to ensure that our clients do not collapse. We have one that is offering computer skills. It is Toughest Young Minds, it is in Sebokeng.’ (P2, female, 29 years)

From the extracts above, the participants seemed to appreciate the availability of rehabilitation centres that they can refer clients to for rehabilitation services. However, it is concerning that clients lack the motivation to use the available facilities. Nonetheless, authors acknowledged that social workers have a duty to assist users of illegal chemical substances in getting to the rehabilitation facilities and facilitate the rehabilitation process (Maina et al. 2021:730).

**Availability of skills development centres:** Some participants liaised with the relevant stakeholders in society, which assists in addressing the substance use crisis and also focuses on out-patients. The following extract indicates some of the available skills development and recovery centres to which the participants can refer clients:

‘Then it means I need to contact the, for example, the Freedom Recovery Centre, because they provide admission for months. If they don’t have space, they will obviously tell me and they will put the client on the waiting list for admission. But I can guarantee you that the waiting list will not be anything more than a month. Then if it is an emergency, then we request on time that “Can you please make space available for this person under these conditions?.”’ (P2, female, 29 years)

As the storyline above attests, the availability of skills development centres is another resource enhancing social workers’ duties within social welfare organisations, However, DeLucia and Solano (2023:30) note that there might not be enough outpatient programmes available to help adolescent substance users, according to treatment services. This could serve as evidence that community-based outpatient treatment facilities for young people struggling with addiction are underfunded for social workers working with adolescents.

**Use and importance of technology:** The availability of tools for service providers is important in the delivery of services to clients. It has been documented in the literature that the social work profession requires items such as computers, cellular phones and printers, amongst other important resources (Filges & Jorgensen 2018:363). Regarding the availability of cellular phones to facilitate consultation, one participant shared the following sentiment:

‘At least we now have cellphones. It used to be a problem as well that one. At least they have improved in that manner, but like I said, now we are still in the ^1^3Gs when the world is at ^2^5G, but, yah, we are going somewhere soon.’ (P1, female, 37 years)

The extract above indicates the need for advanced technology for social workers to get hold of clients using cellular phones, as well as advancing in how meetings and training interventions are held to avoid contact and the spread of the virus (Pascoe 2022:3270). The participant stated that, as social workers, they need to evolve when it comes to technology, as the world is currently using 5G networks, whilst they are provided 3G networks by government.

Therefore, this shows that there is improvement that needs to be taken into consideration in the discipline of social work in terms of technology. According to Bullock and Colvin (2015):

[*T*]echnology has also evolved in social work practice over the past decades, playing a part in giving practitioners easy access to colleagues and to their clients through fax, emails, cell phones, chat rooms, and online messages. (p. 1)

Similarly, the coronavirus disease 2019 (COVID-19) pandemic has also highlighted the importance of technology in the social work profession.

**Financial assistance:** The government, through the DSD, funds several child protection organisations and non-profit organisations. This enables social workers to render adequate services to their clients. The participant below attested to the aid provided by government:

‘Yes. Actually, I think as SANCA [*South African National Drug Dependency and Alcoholism*], we go above and beyond what the Department of Social Development. The money they are giving us; we are even reaching more clients with the money that they are giving us funding for.’ (P7, female, 46 years)

Consistent with the participant’s response, the literature similarly articulates that social workers are allies to public health professionals in efforts to eliminate disparities and improve population health. Consonant with an emphasis by key government institutions on the importance of social welfare, funding (especially from SANCA) is being provided to fight illegal chemical substances by reaching more victims and providing infrastructure to deal with these issues. Furthermore, the degree to which health and social services are financially supported may have an impact on how successfully such services cooperate and coordinate with one another (Best et al. 2017).

## Discussion

Drawing from Watson’s Human Caring Theory, this study sought to explore the gratification social workers receive from working with youth abusing substances. The findings confirm that despite the evidence demonstrating the challenges of this profession, there is also research demonstrating that social workers may find their work fulfilling and experience job satisfaction, believing that they have a positive impact on people’s lives. Social workers are very committed to their job with clients; they have a lot of satisfaction from it, and they believe that they can change people’s lives. The social work profession is premised on the belief that ‘people can become more than they currently are, that their lives hold the possibility of being improved and transformed’ (Yuill & Mueller-Hirth 2019:1533). The participants in this study found gratification in their work by helping their clients sober up, working with their families and bringing meaning back in their lives.

Compassion (Clark & Jen 2023), care and caring (Alacovska 2020) are reciprocal processes that are inherent in the social worker–client relationship. Although the notion of compassion has evolved over time (Garcia-Uribe & Pinto-Bustamante 2024), it essentially reflects a caring attitude towards another person and carries the need to alleviate it (Condon & Makransky 2020). According to Garcia-Uribe and Pinto-Bustamante (2024), compassion ‘represents an appropriate way of being “close” to pain’. Its main objective is to derive an understanding of another person’s suffering. In its etymological sense, the word ‘care’ (*cuidado* as a noun in Spanish) ‘refers to the interest that one puts into something’ (Garcia-Uribe & Pinto-Bustamante 2024:118). Caring is more than kindness; it is deeply involved and consists of meeting as one at the soul or spiritual level (Gunawan et al. 2022). Therefore, it could be argued that ‘compassion is the heart of caring’ (Gustafsson & Hemberg 2022).

Although all the concepts emanate from a community of feelings, compassion is often confused with sympathy and empathy. Etymologically, the word ‘compassion’ stems from the Latin words *com* and *pati*, which mean to suffer with, and are distinct from related constructs such as empathy or kindness (Clark & Jen 2023).

Although this concept was historically founded in religious disciplines, it is widely applicable across many professions. Admittedly, a limited number of studies address compassion specifically within the context of social work practice. However, it remains particularly relevant for the discipline. Compassion is critical for social workers as it demonstrates their care for their clients (Malone & Lewis 2023). Helping others is a source of pleasure and peace for social workers and produces good feelings.

Although the focus of this study was on the gratification social workers receive from working in a substance abuse programme, compassion does not come without caution. Scholars in the psychological literature warn that compassion may threaten social workers’ mental well-being (Jian et al. 2024). Although social workers derive satisfaction from the positive feelings they experience in their caregiving role, prolonged expressions of compassion can come at a price for helping professionals, such as experiencing compassion fatigue (Malone & Lewis 2023). As such, social workers need to be educated and empowered with the necessary tools to identify stressors, become self-aware and participate in self-care activities. The employer should also prioritise the mental health of social workers as its employees. More empirical studies need to be undertaken on how small pockets of success for substance abuse social workers can be coordinated and expanded.

### Limitation

The main limitation of this study, which is typically inherent in a qualitative study, was the relatively small sample. However, this study was not interested in the representation of the population, but in a sample that would provide appropriate and rich insight into the topic. Moreover, the scope of this study was very limited as it addressed very few topics, as outlined under the themes. Lastly, the study was confined to a very small geographical location.

## Conclusion

Despite personal work-related challenges, the study confirmed that some social workers find pleasure in rendering services to the substance use clients. The social workers in this study find gratification in their work by helping their clients sober up, working with their families, and bringing meaning back in their lives. Such work of changing lives encourages them to continue helping to change lives, which, in return, enhances their service delivery. The compassion satisfaction is also encouraged by the support that social workers receive from their supervisors and managers and having the tools of trade available to execute their duties.

## Footnotes

1 The term 3G is referred to as a third-generation mobile network that allows phones, computers and other portable devices to access the internet wherever the device is located.

2 The term 5G is referred to as a fifth-generation mobile network that has high speed, higher upload, more consistent connections and improved capacity as compared to previous mobile networks such as 1G, 2G and 3G.
